# Predictors of alcohol use transitions among drug-using youth presenting to an urban emergency department

**DOI:** 10.1371/journal.pone.0227140

**Published:** 2019-12-31

**Authors:** Jason E. Goldstick, Maureen A. Walton, Amy S. B. Bohnert, Justin E. Heinze, Rebecca M. Cunningham

**Affiliations:** 1 Department of Emergency Medicine, University of Michigan, Ann Arbor, MI, United States of America; 2 Injury Prevention Center, University of Michigan, Ann Arbor, MI, United States of America; 3 Department of Psychiatry, University of Michigan School of Medicine, Ann Arbor, MI, United States of America; 4 Youth Violence Prevention Center, University of Michigan School of Public Health, Ann Arbor, MI, United States of America; 5 Department of Health Behavior and Health Education, University of Michigan School of Public Health, Ann Arbor, MI, United States of America; 6 Hurley Medical Center, Department of Emergency Medicine, Flint, MI, United States of America; Stellenbosch University, SOUTH AFRICA

## Abstract

**Background:**

Precipitants of alcohol use transitions can differ from generalized risk factors. We extend prior research by predicting transitions in alcohol use disorder (AUD) during adolescence and emerging adulthood.

**Methods:**

From 12/2009-9/2011, research assistants recruited 599 drug-using youth age 14–24 from Level-1 Emergency Department in Flint, Michigan. Youth were assessed at baseline and four biannual follow-ups, including a MINI Neuropsychiatric interview to diagnose AUD (abuse/dependence). We modeled AUD transitions using continuous time Markov Chains with transition probabilities modulated by validated measures of demographics, anxiety/depression symptoms, cannabis use, peer drinking, parental drinking, and violence exposure. Separate models were fit for underage (<21) and those of legal drinking age.

**Results:**

We observed 2,024 pairs of consecutive AUD states, including 264 transitions (119 No-AUD→AUD; 145 AUD→No-AUD); 194 (32.4%) individuals were diagnosed with AUD at ≥1 assessment. Among age 14–20, peer drinking increased AUD onset (No-AUD→AUD transition) rates (Hazard ratio—HR = 1.70; 95%CI: [1.13,2.54]), parental drinking lowered AUD remission (AUD→No-AUD transition) rates (HR = 0.53; 95%CI: [0.29,0.97]), and cannabis use severity both hastened AUD onset (HR = 1.18; 95%CI: [1.06,1.32]) and slowed AUD remission (HR = 0.85; 95%CI: [0.76,0.95]). Among age 21–24, anxiety/depression symptoms both increased AUD onset rates (HR = 1.35; 95%CI: [1.13,1.60]) and decreased AUD remission rates (HR = 0.74; 95%CI: [0.63,0.88]). Friend drinking hastened AUD onset (HR = 1.18, 95%CI: [1.05,1.33]), and slowed AUD remission (HR = 0.84; 95%CI: [0.75,0.95]). Community violence exposure slowed AUD remission (HR = 0.69, 95%CI: [0.48,0.99]). In both age groups, males had >2x the AUD onset rate of females, but there were no sex differences in AUD remission rates. Limitations, most notably that this study occurred at a single site, are discussed.

**Conclusions:**

Social influences broadly predicted AUD transitions in both age groups. Transitions among younger youth were predicted by cannabis use, while those among older youth were predicted more by internalizing symptoms and stress exposure (e.g., community violence). Our results suggest age-specific AUD etiology, and contrasts between prevention and treatment strategies.

## Introduction

Alcohol use disorder (AUD) refers to a dysfunctional drinking pattern leading to clinically significant symptoms of physical or psychological distress [1] and is highly prevalent in the US, with estimated twelve month and lifetime prevalence of 13.9% and 29.1%, respectively [2]. AUDs confer substantial medical risks—as alcohol use causes over 30 medical conditions, and increases risk of developing several others [3]—and are associated with increased disability [4] and lowered quality of life [5]. In addition, excessive alcohol use increases risk for other behavioral comorbidities that have their own associated health risks, including violence involvement [6–9], motor vehicle crash [10], sexual risk behaviors [11, 12], and other drug use [13, 14]. Determining predictors of transitions in AUD status (e.g., AUD onset, AUD remission) is crucial for intervention design, as it provides a basis for tailoring interventions to AUD primary prevention and to AUD treatment.

While prior research has shown that factors like depression and anxiety [15, 16], peer influences [17–20], parental influences/practices [21, 22], and community-level factors [23–25] are important aspects of the etiology of alcohol use, little has focused on how those factors modulate transition rates between levels of alcohol use throughout adolescence and emerging adulthood. Some researchers have recognized that precipitants of alcohol use transitions across youth development can differ from generalized risk factors for problem drinking. For example, some researchers suggest that social factors play a larger role in alcohol use initiation, whereas long term problematic alcohol use is driven more by individual factors, such as depression and anxiety [26]. Little research has empirically addressed such those hypotheses, but one study analyzing the time between two alcohol use benchmarks (e.g., first drink to first AUD diagnosis) using Cox regression showed the importance of social factors in the escalation of drinking, and that internalizing symptoms were only a risk factor for progression to AUD [27]. We seek to build upon that work by modeling transitions into—and out of—AUD within a unified framework that allows for explicit testing of whether predictors of AUD development differ from those of continued AUD. Focusing on predictors of transitions—as opposed to general risk factors for problem drinking—will help identify intervention content that depends on current alcohol use level, as well as factors that may be effective regardless of current use level.

Identifying relevant intervention content is especially important in populations that are both at most need, and potentially the most amendable to intervention. Youth entering the emergency department (ED)—particularly for a violent injury—are at high risk for substance use [28]. Such youth also frequently have other mental health service needs [29], are at elevated risk of future violent injury [30], and are more likely to be involved in other high-risk behaviors, such as firearm violence [31, 32]. The ED is a critical intervention site both because it provides an opportunity to reach youth that may not be reachable through schools or the criminal justice system [33], and because an ED visit is a “teachable moment” where individuals may be more amendable to intervention, including alcohol use interventions specifically [34]. Taking advantage of this opportunity requires precise focus on the most relevant intervention content in this population.

We examined data from a prospective cohort study of drug-using youth presenting to an urban ED to determine the prevalence of AUD in this population, and the frequency of AUD transitions. We further examined how those transition rates are modulated by individual-, social-, and community-level factors including demographics, other substance use, mental health symptoms, peer and parental behaviors, and violence exposure. Given known age-specific changes in the rates and reasons for alcohol use [35], as well as issues surrounding alcohol availability among underage youth, we hypothesized that some factors driving AUD transitions may differ with age and therefore analyzed predictors of AUD transitions in underage youth (<21) and those of legal drinking age separately. Our a priori hypothesis was that AUD initiation would be driven more by social factors, but sustained AUD would relate more to internalizing symptoms, and external stressors such as violence exposure.

## Methods

### Study setting

The Flint Youth Injury (FYI) study was conducted at Hurley Medical Center (HMC) in Flint, Michigan beginning in December 2009. HMC is the only Level-1 trauma center in Flint and has an annual patient census of ~100,000, and has been the setting for prior research studies [36, 37]. The demographics and crime rates in Flint are comparable to that of other legacy cities such as Camden, NJ. Our study population is a majority Black (~58%), reflecting the racial makeup of Flint.

### Ethics statement

FYI study staff obtained informed consent—or assent, with parental consent for youth under 18—for all youth screened, and consent (or assent with parental consent) was obtained again from all eligible youth prior to participation in the longitudinal study. An NIH certificate of confidentiality was obtained for the FYI study. The Institutional Review Boards (IRBs) of both Hurley Medical Center and the University of Michigan reviewed and approved all study protocols.

### Study design and procedures

The purpose of the FYI study was to evaluate the medical and mental health service needs of drug-using youth age 14–24 seeking medical care at HMC, and compare the service needs of violently injured (VI) youth with a non-violently-injured comparison group (CG). In service to that, research assistants recruited VI youth for the FYI study from December 2009 to September 2011, ≥21hrs/day (only 5am-2am on Tuesday and Wednesday), 7 days per week. For each VI youth approached, research assistants approached the next non-VI youth falling into the same age group (14–17, 18–20, 21–24) and sex for screening. Youth screening positive for any past-six-month drug use were eligible for the cohort study. Exclusions included inability to consent (e.g. due to mental or physical incapacitation), and ED presentation for child abuse or sexual assault. Research assistants approached initially unstable patients if they stabilized within 72 hours.

Staff administered surveys via tablet to eligible youth who entered the longitudinal study at baseline, and every six months for two years, comprising five assessments in total. Youth were remunerated $20 for the baseline assessment, and $30, $40, $45, and $50 for each of the four respective follow-ups. Attrition was minimal, with ≥83.7% of youth assessed at each follow-up; methods used to achieve those follow-up rates are described elsewhere [38].

### Measures

#### Alcohol use disorder (AUD)

At each assessment, youth were administered the Mini-International Neuropsychiatric Interview (MINI) [39]. Current DSM-IV diagnoses of alcohol abuse and current alcohol dependence were made using the MINI. Current AUD was defined as either current abuse or dependence. Throughout we will use the nomenclature of “AUD onset” to refer to No AUD → AUD transitions, “AUD remission” to refer to AUD → No-AUD, and “AUD persistence” to refer to AUD → AUD “transitions”.

#### Demographics

Participants answered questions from the DATOS [40] and National Longitudinal Study of Adolescent Health [41] regarding demographic characteristics including age, sex, race, and receipt of public assistance. We collapsed race to an indicator of Black race, given that over 90% of the participants self-reported as either Black or White. Public assistance was an indicator of any current receipt of public assistance by the participant or their parents.

#### Cannabis use and mental health

The NIDA modified Alcohol, Smoking, and Substance Involvement Screening Test (ASSIST) [42] measured past-six-month cannabis use; we used the mean of the six cannabis ASSIST items to quantify cannabis use severity. Because other substance use was relatively infrequent in this population, we did not include other substance use measures. The Brief Symptom Inventory (BSI) [43] measured current depression and anxiety symptoms; we used the means of the six depression and the six anxiety BSI items to quantify severity of the respective symptoms.

#### Social exposures

We measured peer and parental exposures, including both support and alcohol use, using validated instruments. The social support scale of Procidano and Heller measured parental support [44], and a question from the Flint Adolescent study measured parental alcohol use frequency [45]. Items from the Flint Adolescent Study [46] measured both positive peer exposures (e.g. number of friends receiving good grades in school), and peer alcohol use frequency; the latter was a single item, and the former was the average of four items.

#### Violence exposure

We measured violence victimization and community violence exposure using validated instruments. Twenty-six items (thirteen partner; thirteen non-partner) from the modified Conflict Tactics Scale [47] measured partner and non-partner physical violence victimization ranging from non-severe (e.g., slapping/hitting) to severe (e.g., weapon violence). We reduced those measurements to two indicators—any partner victimization (yes/no) and any non-partner victimization (yes/no). Five items from the “Things I’ve Seen and Heard” survey measured community violence exposure (e.g. “I have seen somebody shot or stabbed”); we averaged those items to produce a summary score of community violence exposure.

### Statistical analysis

**Fig 1:**
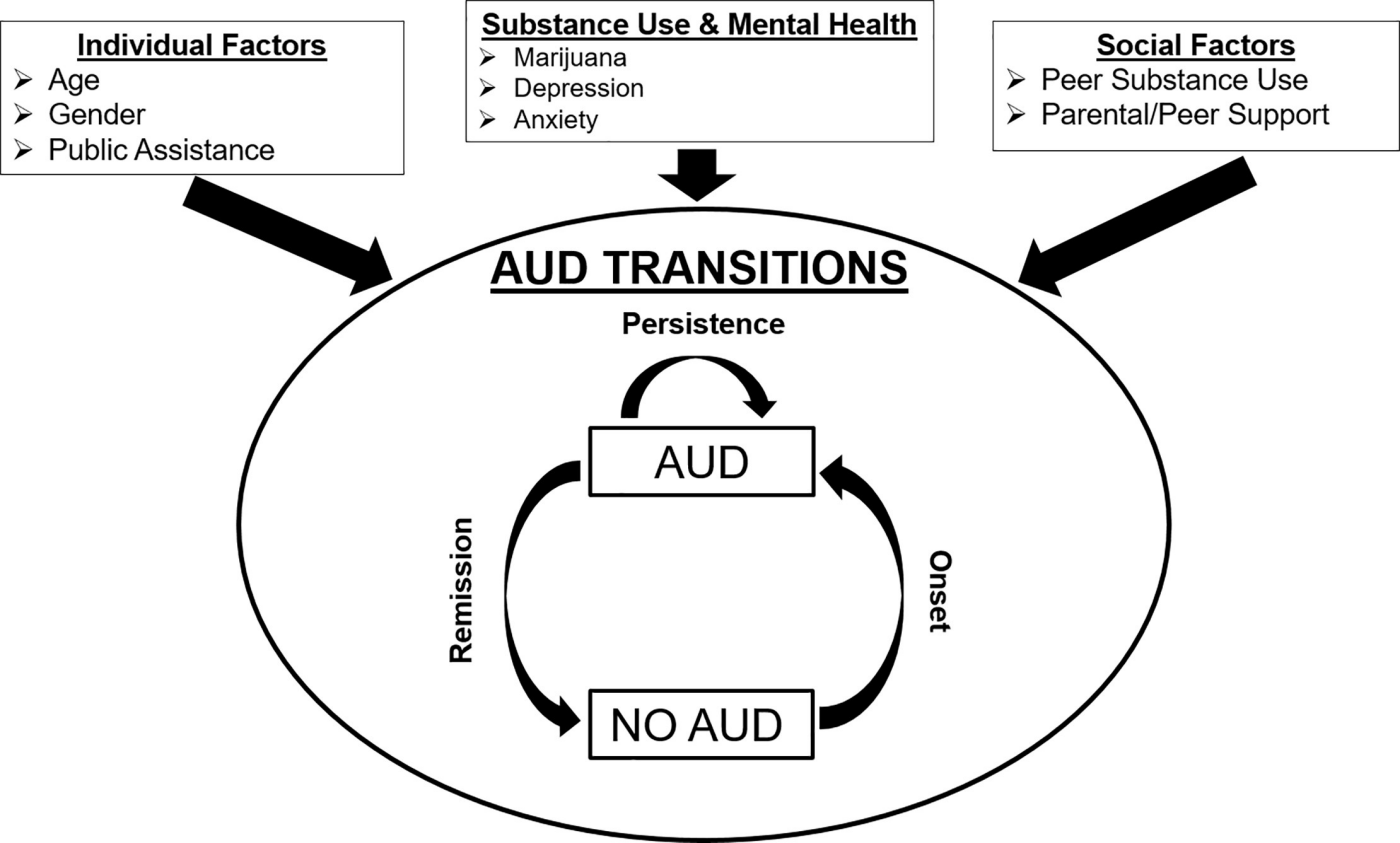
Conceptual model of AUD transitions.

The overall analytic goal was to determine rates of AUD and AUD transition among our study population, and to identify covariates that predicted transitions in AUD—both in terms of facilitating AUD onset (i.e. No-AUD → AUD transitions) and AUD remission (i.e. AUD → No-AUD). The conceptual model underlying this analysis is shown in Fig 1. We began by calculating frequencies of AUD throughout the course of the study in each age group, and the number of AUD transitions among underage youth (<21 years) and those of legal drinking age. We then descriptively compared transitions, e.g., among those with current No-AUD, we contrasted those who transitioned to AUD at the next time point with those who did not.

Our primary modeling approach was to model current AUD status as a continuous time inhomogeneous Markov Chain [48], with covariate-dependent transition probabilities. Within these models, the transition rate between two states (i.e., “No-AUD” and “AUD”) is parameterized in terms of the transition intensity function, which is interpreted similarly to the hazard function in survival analyses, without the presence of a “death” state (i.e., individuals can transition back and forth between states). The transition intensity between two states (e.g., *i*: No-AUD; *j*: AUD) is assumed to depend on the pre-transition covariate values (*z*) through a log-linear model:

$$\log(q_{ij})=\alpha_{ij}+\beta_{ij}z$$

Under this model, the covariate values and the time-lag determine the transition probabilities. The exponentiated regression coefficients estimated from this model are interpreted as hazard ratios (HRs)—showing the effect the covariate on the transition rate between two states. Because of developmental differences in rates of use, reasons for use, and alcohol availability, we fit separate models for underage (<21 years) youth and those of legal drinking age. The R package *msm* was used for model fitting [48].

Missing data and differential follow-up times are handled internally under this model. Because the transition probabilities only depend on the covariate values and the time gap between measurements, transitions occurring during missed follow-ups can still be incorporated into the model. For example, if an individual completes a baseline (say, on 1/1/2010), misses the 1^st^ follow-up, and completes the 2^nd^ follow-up on 1/20/2011, that pair of measurements taken 12.7 months apart can be incorporated into the model without assuming anything about that individual’s would-be state at the 1^st^ follow-up. Relatedly, although subjects were ascertained on approximate 6-month schedules, there is small heterogeneity in the exact follow-up times; this model does not require the assumption that all time lags are equivalent.

To build the models, we began by individually testing the “state-dependence” of the effect of each covariate that showed significant unadjusted effects in the descriptive contrasts between those who did versus did not transition (with the exception of demographic control variables, which were included regardless of significance). We tested each covariate effect’s “state-dependence” (i.e. whether a predictor’s effect on AUD onset risk differs from its effect on AUD persistence) by constraining the hazard for future AUD to be independent of the current state (in terms of the model formulation, this amounts to testing the null hypothesis that *β_ij_* = −*β_ji_*). If there was little evidence (p > 0.20) of state-dependence, we constrained the effects to be equivalent in the adjusted models—effectively borrowing information across states (“AUD” and “No-AUD”) to estimate covariate effects. We use the lower *p*-value threshold to conclude state-dependence to avoid imposing a constraint that effectively nullifies the “transition” component of the model, unless there is very little evidence against it—particularly given the age stratification in this analysis and the accompanying reduction in the power of the state-dependence test. When appropriate, that constraint lessens the power issues brought about by the relatively small numbers of transitions in some cases. In the final models, covariate effects were constrained to be equivalent across states, except those showing evidence of state dependence.

## Results

### Participants

In total, 1,448 youth were screened (718 VI) among which 666 (388 VI) were eligible for the longitudinal study. Among those, 599 (349 VI) consented to the longitudinal study and completed the baseline. The baseline sample was majority male (n = 352; 58.8%), majority Black (n = 349; 58.3%), and majority on public assistance (n = 437; 73.0%). A large majority reported past 6-month cannabis use at baseline (n = 583; 97.3%). The full study flow chart has been published previously (26, 28).

### Descriptive analysis

**Fig 2:**
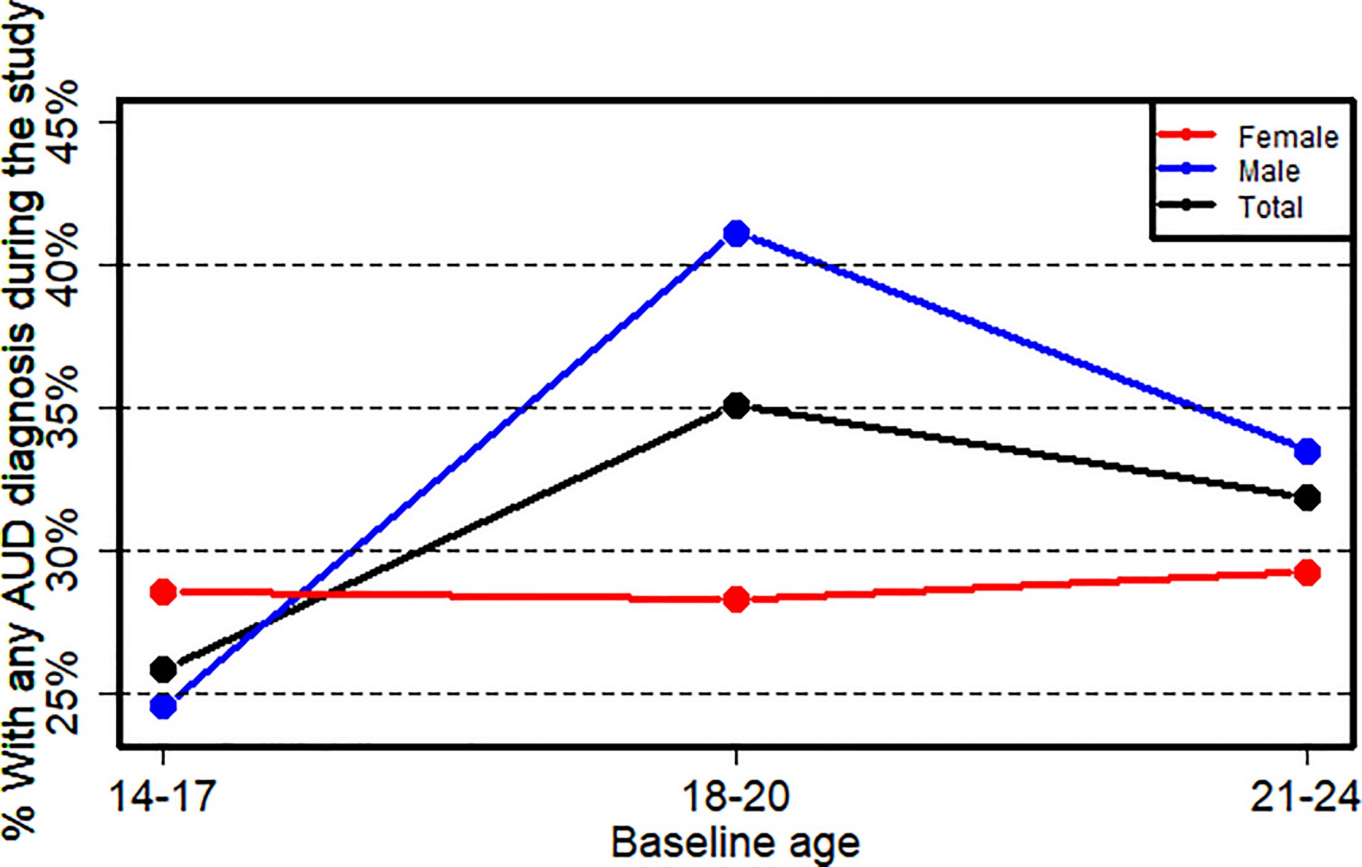
Prevalence of any AUD during the study by sex and age group.

Among the 599 youth followed prospectively, 2,631 diagnostic interviews were conducted, resulting in a total of 352 diagnoses (13.3% of person/time points). In total, 194 individuals (32.4%) had an AUD diagnosis at ≥1 follow-up, including 34.9% of males (n = 123) and 28.7% of females (n = 71). Fig 2 shows the proportion with any AUD during the study, by sex and age group (14–17, 18–20, 21–24). Among females, the prevalence of any AUD during the study was just under 30% across age groups; males in the 18–20 age group had 2/3 higher risk of AUD during the study than those in the 14–17 age range (Fig 2). Among underage youth, there were 54 and 55 transitions from No AUD to AUD (onset), and from AUD to No AUD (remission), respectively. Among youth of legal drinking age, there were 56 and 71 transitions from No AUD to AUD (onset), and from AUD to No AUD (remission).

Table 1 shows comparisons between those who did and did not transition AUD status at two consecutive measurements, stratified by age group (14–20, 21–24) and state at the preceding time point (No AUD, AUD). Among underage youth, AUD onset was more common among males and non-Black youth, and were more likely to be preceded by higher levels of friend drinking, community violence exposure, and cannabis use. Underage youth currently diagnosed with AUD were less likely to achieve remission (i.e. transition to No AUD) if they were older, reported higher levels of mental health symptoms and cannabis use, and reported higher levels of parental drinking. Among youth of legal drinking age, AUD onset was more common among males and non-Black youth, and was more likely to be preceded by higher levels of friend drinking and anxiety symptoms; similar trends appeared with depressive symptoms, but those trends were not statistically significant (p = 0.09). Youth age 21–24 currently diagnosed with AUD were less likely to achieve remission if they had more anxiety and depression symptoms, and if they reported higher levels of friend drinking and community violence exposure.

**Table 1:** Descriptive comparisons between different AUD transition types among children and among adults.

	No AUD→No AUD	No AUD→AUD	AUD→No AUD	AUD→AUD
Age 14–20				
Number	661	54	55	43
Male	**354 (53.5%)**	**37 (68.5%)**	34 (61.9%)	24 (55.8%)
Age	18.26 (1.36)	18.17 (1.38)	**18.15 (1.33)**	**18.74 (1.09)**
Black	**439 (66.4%)**	**27 (50.0%)**	24 (43.6%)	12 (27.9%)
Public Assistance	494 (74.7%)	37 (68.5%)	42 (76.4%)	36 (83.7%)
Anxiety (BSI)	0.46 (0.71)	0.55 (0.78)	**0.69 (0.87)**	**1.22 (1.16)**
Depression (BSI)	0.62 (0.78)	0.79 (0.95)	**0.82 (0.81)**	**1.28 (1.05)**
Partner Victimization	323 (48.9%)	28 (51.9%)	34 (61.9%)	29 (67.4%)
Non-Partner Victimization	292 (44.2%)	24 (44.4%)	39 (70.9%)	32 (74.4%)
Parental Drinking	1.45 (0.66)	1.43 (0.54)	**1.56 (0.79)**	**1.96 (0.62)**
Parental Support	3.26 (1.23)	3.06 (1.22)	2.92 (1.34)	2.98 (1.23)
Friend Drinking	**2.23 (1.03)**	**2.81 (1.07)**	3.31 (1.17)	3.51 (1.22)
Friend (+) Influence	2.23 (0.74)	2.25 (0.60)	2.10 (0.78)	2.08 (0.88)
Community Violence	**1.56 (0.87)**	**1.81 (0.88)**	2.07 (0.90)	2.04 (0.93)
Cannabis Use	**1.62 (1.35)**	**2.29 (1.36)**	**2.26 (1.50)**	**2.89 (1.50)**
Age 21–24				
Number	857	56	71	64
Male	**485 (56.6%)**	**42 (75.0%)**	47 (66.2%)	35 (54.7%)
Age	22.68 (1.42)	22.88 (1.57)	22.79 (1.44)	22.97 (1.31)
Black	**531 (62.0%)**	**25 (44.6%)**	38 (53.5%)	36 (56.3%)
Public Assistance	641 (74.8%)	39 (69.6%)	51 (71.8%)	44 (68.8%)
Anxiety (BSI)	**0.49 (0.79)**	**0.90 (1.02)**	**0.90 (0.89)**	**1.44 (1.00)**
Depression (BSI)	0.64 (0.85)	0.86 (0.94)	**1.03 (0.98)**	**1.43 (1.00)**
Partner Victimization	321 (37.4%)	25 (44.6%)	41 (57.7%)	41 (64.1%)
Non-Partner Victimization	271 (31.6%)	17 (30.4%)	39 (54.9%)	41 (64.1%)
Parental Drinking	1.50 (0.76)	1.58 (0.79)	1.76 (0.99)	1.73 (1.03)
Parental Support	3.08 (1.34)	2.87 (1.37)	2.86 (1.32)	2.53 (1.24)
Friend Drinking	**2.47 (1.09)**	**3.02 (1.17)**	**3.14 (1.29)**	**3.58 (1.27)**
Friend (+) Influence	2.10 (0.71)	2.08 (0.69)	2.11 (0.73)	1.97 (0.71)
Community Violence	1.34 (0.91)	1.40 (0.81)	**1.86 (0.90)**	**2.20 (0.78)**
Cannabis Use	1.43 (1.32)	1.62 (1.46)	2.31 (1.57)	2.60 (1.54)

Note: Entries are counts (%) for categorical variables and mean (SD) for numeric variables. Bold indicates p < 0.05

### Markov Chain models

Based on unadjusted significance we included depressive/anxiety symptoms, parental drinking, friend drinking, community violence exposure, and cannabis use in the age 14–20 model, and we included depressive/anxiety symptoms, friend drinking, and community violence in the age 21–24 model. We also included age, sex, race, and public assistance in both models. Due to collinearity and similarity in their effects, we combined anxiety and depressive symptom scores by averaging the two.

Table 2 shows the unadjusted hazard ratios and the p-value testing “state dependence” in each variable’s effect—i.e. whether its effect on future AUD is different based on presence of a current AUD diagnosis. Among youth age 14–20, age, anxiety/depressive symptoms, and parental drinking had larger effects among those with current AUD, while male sex, and friend drinking showed some evidence (p < 0.20) of larger effects among those without current AUD. Among those age 21–24, male sex and non-Black race both were larger risk factors for future AUD among those without current AUD, and community violence exposure was only a risk factor for AUD persistence. No other variables showed evidence of state dependence.

**Table 2:** Unadjusted covariate state-dependence tests among variables in the final model.

	HR (No AUD→ AUD)	HR (AUD→No AUD)	*p*
Age 14–20			
Age	0.83 (0.65, 1.07)	0.73 (0.58, 0.93)	0.02
Male	2.04 (1.08, 3.88)	1.24 (0.68, 2.26)	0.08
Black	0.64 (0.34, 1.21)	1.55 (0.84, 2.89)	0.99
Public Assistance	0.65 (0.32, 1.31)	0.70 (0.34, 1.47)	0.21
Depressive/Anxiety Symptoms	1.05 (0.73, 1.52)	0.62 (0.42, 0.90)	0.16
Parental Drinking	0.78 (0.49, 1.23)	0.47 (0.28, 0.79)	0.01
Friend Drinking	1.51 (1.15, 1.99)	0.95 (0.71, 1.27)	0.15
Community Violence	1.38 (0.97, 1.96)	1.02 (0.73, 1.42)	0.25
Cannabis Use	1.26 (1.03, 1.55)	0.81 (0.65, 1.01)	0.91
Age 21–24			
Age	1.08 (0.89, 1.31)	0.94 (0.78, 1.12)	0.95
Male	2.56 (1.34, 4.89)	1.39 (0.82, 2.36)	0.008
Black	0.49 (0.27, 0.88)	0.99 (0.59, 1.65)	0.12
Public Assistance	0.81 (0.43, 1.52)	1.09 (0.63, 1.91)	0.82
Depressive/Anxiety Symptoms	1.26 (0.94, 1.56)	0.65 (0.47, 0.91)	0.49
Friend Drinking	1.36 (1.08, 1.73)	0.83 (0.67, 1.01)	0.53
Community Violence	0.88 (0.63, 1.24)	0.66 (0.48, 0.92)	0.06

Note: p is the p-value corresponding to a test of whether the HR from No-AUD (column 1) differs from the inverse of the HR from AUD (column 2), which would indicate that risk factors for future AUD differ based on current AUD status.

Table 3 shows the final Markov Chain model for each age group. To test model fit we used a Pearson-type lack-of-fit test based on contrasting observed with model-predicted transition rates, with p-values estimated by 1000 bootstrap iterations [49]; there was no evidence of lack-of-fit in the age 14–20 model (p = 0.39) or the age 21–24 model (p = 0.51). Among those age 14–20, males experience AUD onset at more than twice the rate of females, and the rate of AUD remission was 29% lower for each additional year of age. Greater cannabis use severity both hastened AUD onset (18% increase in the rate for each ASSIST point) and slowed AUD remission (15% slower rate for each ASSIST point) among underage youth. Peer drinking frequency increased rates of AUD onset, and greater parental drinking lowered rates of AUD remission among those age 14–20. Among those age 21–24, males had nearly 3x faster rates of AUD onset than females. Depression/anxiety symptoms and friend drinking frequency both increased rates of AUD onset and decreased rates of AUD remission among those age 21–24. Community violence exposure lowered AUD remission rates among those of legal drinking age. No other effects were statistically significant in the adjusted models.

**Table 3:** Adjusted Markov Chain transition model for transitions between FA states.

	HR (No AUD→ AUD)	HR (AUD→No AUD)
Age 14–20		
Age	0.83 (0.61, 1.14)	**0.71 (0.53, 0.95)**
Male	**2.28 (1.03, 5.08)**	1.57 (0.66, 3.74)
Black†	0.73 (0.54, 1.00)	1.36 (1.00, 1.86)
Public Assistance†	0.87 (0.62, 1.23)	1.14 (0.81, 1.61)
Depressive/Anxiety Symptoms	0.95 (0.60, 1.50)	0.76 (0.46, 1.27)
Parental Drinking	0.62 (0.37, 1.04)	**0.53 (0.29, 0.97)**
Friend Drinking	**1.70 (1.13, 2.54)**	1.26 (0.83, 1.93)
Community Violence†	1.04 (0.86, 1.25)	0.96 (0.80, 1.16)
Cannabis Use†	**1.18 (1.06, 1.32)**	**0.85 (0.76, 0.95)**
Age 21–24		
Age†	1.06 (0.96, 1.17)	0.94 (0.85, 1.04)
Male	**3.00 (1.47, 6.15)**	1.13 (0.61, 2.10)
Black	0.57 (0.29, 1.11)	1.05 (0.58, 1.88)
Public Assistance†	0.86 (0.63, 1.18)	1.16 (0.85, 1.58)
Depressive/Anxiety Symptoms†	**1.35 (1.13, 1.60)**	**0.74 (0.63, 0.88)**
Friend Drinking†	**1.18 (1.05, 1.33)**	**0.84 (0.75, 0.95)**
Community Violence	0.77 (0.53, 1.12)	**0.69 (0.48, 0.99)**

Note: Bold indicates p < 0.05

†: Effects are constrained across states so that the HR for transitions out of No-AUD is equal to the inverse of the HR out of AUD, corresponding to forcing risk factors for AUD to have equivalent effects regardless of whether the transition is from No-AUD or AUD.

## Discussion

This study provides a novel characterization of previously unknown rates, and predictors, of transitions in AUD diagnosis among drug-using youth presenting to an urban ED. Nearly a third of such youth were diagnosed with AUD during the study period, and transitions in AUD—both in terms of onset, and remission, of AUD—were common. Those transitions could be predicted by both static and modifiable factors, suggesting avenues for both primary prevention intervention content, and intervention content for those currently with AUD. Among youth below legal drinking age, development of AUD was preceded by higher rates of friend drinking, and sustainment of AUD was preceded by greater parental drinking; other substance (cannabis) was a risk factor among both those with and without current AUD diagnosis. Among youth ≥21 years old, friend drinking and internalizing (depression/anxiety) symptoms increased both AUD development and persistence among youth of legal drinking age, while other stressors such as community violence exposure were particularly risky for AUD persistence. Across age groups, males had much higher rates of AUD onset, but not higher rates of AUD persistence. These results identify content that potentially prevents AUD across current levels of alcohol use, as well as content that is specific to current AUD status, in each age group. Integrating this information may help optimize the teachable moment presented by an ED visit for prevention.

Higher self-reported friend drinking frequency was broadly predictive of future AUD across age groups, consistent with prior research on the importance of peer behavior on youth drinking [9, 50]. The fact that reports of high friend drinking preceded AUD in both age groups suggests this association doesn’t arise purely from peer selection. Our results may arise from direct alcohol use exposure in social situations, or from effects arising from perceived drinking norms. Parental drinking may also contribute to both factors; the fact that parental drinking only appeared as a risk factor for underage youth AUD persistence may result from greater parental cohabitation rates among that age group. Regarding perceived drinking norms, researchers have found that youth perceptions of peer drinking are often exaggerated [51], suggesting the utility of normative resetting interventions, which are evidenced-based among college students [52, 53]. Combining such approaches with strategies—such as motivational interviewing—to empower youth to prevent problem drinking in the context of social alcohol exposure may be an important universal prevention strategy.

Internalizing symptoms such as anxiety and depression were important components of AUD onset and persistence among youth of legal drinking age, but not among underage youth. This is consistent with prior literature identifying a positive association between alcohol use and depression and anxiety symptoms [15, 16]. The present study further suggests an important distinction in AUD epidemiology between the two age groups. One possible explanation for this age-specific association may suggest that AUD develops—and persists—among emerging adults in an attempt to cope with negative affect, which is also consistent with the community violence exposure finding that was specific to older youth. This is consistent with prior research showing that acceleration of binge drinking in youth age 18–22 was correlated with boredom/desire to get high alcohol use motives, while continuation of binge drinking among older emerging adults (age 22–30) was associated with drinking motivated by the desire to escape from problems [54]. AUD prevention strategies among older youth in our target population may benefit from added emphasis on coping and resiliency strategies. Interventions have been effective at improving coping and self-esteem in the context of alcohol use prevention [55], and our results suggest those approaches may be especially important in preventing and treating AUD among emerging adults in this population.

Cannabis use emerged as a predictor of both AUD onset and AUD persistence among underage youth, but not youth ≥21 years old, in this population. This finding, combined with the importance of social exposures, and the lack of findings with regard to internalizing symptoms and community violence exposure, may suggest that AUD arises primarily as a social process, and an escalation of other substance use, among underage youth in this population. One explanation could be related to the fact that at the time this data was collected, cannabis availability may be less age-specific in this population than alcohol, which becomes increasingly available with age; however, this relationship could change given trends towards recreational cannabis legalization. Regardless, findings suggest a focus on cannabis users for AUD prevention programs, and a combined emphasis on both cannabis and alcohol use among youth with AUD. Multi-behavioral interventions have been effective in other ED-based research [56], and may be especially important to incorporate into youth AUD interventions.

We showed that males have higher rates of AUD in this population than females, which is consistent with prior research in nationally representative samples [57]. The new information provided by this work is that this finding is a) primarily seen with regard to AUD development, and not sustainment; and b) it is consistently seen in both underage youth and emerging adults of legal drinking age, with males showing over twice the rate of AUD onset among both groups. Those results suggest that, although males have higher rates of AUD onset, after AUD has been developed, it is sustained similarly in both sexes, and this is true across age groups. Thus, males should be considered high-risk groups for AUD primary prevention programs, regardless of age, but are not necessarily so for treatment programs among those with a current AUD diagnosis. Although unadjusted findings did suggest non-Black youth were at higher risk for AUD development, this did not hold in the adjusted findings. Regardless, culturally competent intervention strategies are critically important, and have been found more effective with regard to substance use interventions [58].

We acknowledge limitations of this work. The first is that this study took place at a single site. Although, this site is generally comparable to urban EDs in other legacy cities, additional studies with longitudinal ascertainment of AUD and relevant predictors are necessary to understand similarities and differences across study populations. Second, we do not have access to diagnosis history prior to this study, so we cannot know whether transitions into AUD represent the true initiation of AUD in that individual, or whether it is a recurrence of prior AUD. However, this limitation does not diminish the importance of predicting transitions in AUD from one time point to the next. Third, this analysis focused on a study population reporting substance use at baseline, which comprises about half of youth seeking care at the ED [29]. Although the demographics of our sample mirror those of youth in the city of Flint, our study population may be at particularly high risk for substance use and related problems. We note that a large majority of the baseline substance use was cannabis use, and that there was notable variability in the severity of cannabis use, making its use as a predictor still feasible. Exploration of populations not selected based on drug use is still required. Finally, more detailed measures of mental health symptom severity, substance co-use, external stressors, and coping strategies could be important predictors; future studies with such measurements could provide important supplements to this work.

Despite of those limitations, our study provides previously unavailable information about the predictors of AUD transitions across two key developmental age groups. Emphasis on friend influences are broadly important for AUD prevention across age groups, which may take the form of modifying norms or resiliency in the face of friend drinking. Among underage youth, emphasis on other substance use is important regardless of current AUD status, while emerging adults (≥21 years) may universally benefit from greater emphasis on treating internalizing symptoms such as anxiety and depression. A focus on more proximal social factors—such as parental drinking—may be more important for underage youth already diagnosed with AUD. Similarly, emerging adults (ages 21+) already diagnosed with AUD may require content related to treating environmental stress arising from factors like community violence. By paying attention to factors preceding transitions, we identified strategic targets for content specific to prevention, and that specific to intervention, in two age groups—this information can improve the efficiency of behavioral intervention opportunities in clinical settings.
